# Molecular Dynamics vs. Stochastic Processes: Are We Heading Anywhere?

**DOI:** 10.3390/e20050348

**Published:** 2018-05-07

**Authors:** Giovanni Ciccotti, Mauro Ferrario, Christof Schütte

**Affiliations:** 1 Institute for Applied Mathematics “Mauro Picone” (IAC), CNR, Via dei Taurini 19, 00185 Rome, Italy; 2 University of Rome “La Sapienza”, P.le Aldo Moro 5, 00185 Rome, Italy; 3 University College Dublin (UCD), Belfield, Dublin 4, Ireland; 4 Dipartimento di Scienze Fisiche, Informatiche e Matematiche, University of Modena and Reggio Emilia, Via Campi 213/A , 41125 Modena, Italy; 5 Institut für Mathematik, Freie Universität Berlin and Zuse Institute Berlin (ZIB), Takustr. 7, D-14195 Berlin-Dahlem, Germany

In recent decades, molecular simulation has developed into an industry. Since its beginnings in the 1950s, the field has grown continuously with the tremendous increase of computational power but, even more, by the successful efforts of many researchers devoted to finding ways of computing the properties of aggregates of molecular systems at atomistic resolution. Molecular simulations developed from basic theoretical techniques (Molecular Dynamics, MD, and Monte Carlo. In the following we will focus on MD although for many problems the two techniques go arm in arm) to compute all kinds of properties of condensed phases for the simplest molecular models. For a long time, these calculations could be done only for simple systems, but computing power and algorithms began catching up with the theory to enable the application of calculations to large, practically relevant molecules like proteins, drugs and materials. In 1998 the Nobel Prize in chemistry was awarded to Walter Kohn and John A. Pople for the development of advanced computational methods in quantum chemistry while in 2013 it was awarded to Martin Karplus, Michael Levitt and Arieh Warshel for the development of models for complex chemical systems, so confirming that the field has reached a “nobility status”. If you ask a professional in the field: “Where is MD heading next?”, the answer is generally “larger, faster, more complex”: The field seems obsessed with gearing up the machinery for advancing to more complex, and more relevant molecular systems. This may be an appropriate target but, as a general answer, it is also a treacherous one. It reveals that MD as a scientific field has changed scope and purpose. MD started as an auxiliary tool of the theory that made possible calculations that scientists could not even dream of doing by hand before. It started as a simple prolongation of the human mind. Then, step by step, the machinery was scaled up and simulations started to allow realistic insight into the molecular basis of the cosmos and also into the molecular micro-cosmos itself. These simulations seem to create a virtual reality that scientists just have to visit in order to find out “what is going on”. Furthermore, today, at the onset of the era of exascale computing, the dream of inspecting the processes of life on the level of molecular resolution seems to be coming true. However, there are pitfalls. For example, even in the exascale era, clock speed and bandwidth will no longer substantially increase. That is, the more complex molecular systems that come into reach will be larger but the timescale accessible by brute-force MD simulations will no longer grow. This means that the timescale challenge will not be solved by the next generation of supercomputers but only by theory. Since the beginning, research regarding algorithms to overcome the challenges associated to the limitations of the atomistic approach have been at the heart of the development of the field. However, in recent years, the pace of the phenomenon, specially focused on the timescale challenge, has witnessed a dramatic acceleration. Ideas from statistical physics, rare event simulations, and related fields, generally led by stochastic modelling, have been utilized for new algorithms like milestoning, interface sampling, Markov state modelling, enhanced sampling or accelerated MD that allow for obtaining reliable statistics on timescales far beyond the ones accessible by the longest MD trajectories computable by brute force. At the same time, the gap between simulation and understanding in molecular dynamics is widening. One reason for this gap is that massive simulations produce massive data and tools for understanding it have not developed and are not developing at the same rate as the rate of increase in simulation size. Simultaneously, fields like machine learning and big data analytics are experiencing explosive growth. The idea of using the progress in the data sciences for the extraction of information from long MD trajectories is not new; however, recently, many research groups have taken the lead and developed completely new techniques, e.g., for data-based identification of reaction coordinates or kinetic fingerprinting, that are simultaneously based on simulation as well as on experimental (spectroscopic) data. However, again, theory is needed to provide understanding of what is actually computed and with which accuracy. In this promising but complex situation, the standard trial and error way in which the field has progressed in the past, led by chemists and physicists not particularly mathematically (probabilistically) trained, seems to have reached some kind of saturation and more attention to the tools of applied mathematics appears to be needed. In particular, if we aim beyond standard statistical mechanics and equilibrium expectation values at dynamical phenomena. Developing the right probabilistic framework for the study of dynamical phenomena, such as rare events or transition pathways of non-equilibrium processes, is a formidable challenge. This part of Statistical Mechanics is still much less developed and requires more sophisticated tools from Stochastic Processes Theory; one has to deal with a stochastic process rather than with random variables. As a result, the probability distributions relevant to dynamical phenomena are more complicated objects in path space, and their theoretical characterization and efficient computation are in their infancy. However, establishing what these distributions are and how to use MD or modifications of it as a tool to sample them efficiently is the right way to go. Given the growing importance of advanced statistical techniques and stochastic modeling to understand and develop a more solid basis for computational statistical mechanics and, in particular, MD simulation, we need a novel effort to overcome the traditional way of approaching this problem. The present Special Issue is a first attempt to collect some of these new theory-related research efforts in the framework of MD, specially addressing algorithms [1,2,3,4], theoretical methods [5,6,7] and rigorous mathematical formulations [8,9]. The issue also presents some further applications [10,11] of molecular dynamics which can be of use while widening the perspective.
